# DBM-DB: the diamondback moth genome database

**DOI:** 10.1093/database/bat087

**Published:** 2014-01-16

**Authors:** Weiqi Tang, Liying Yu, Weiyi He, Guang Yang, Fushi Ke, Simon W. Baxter, Shijun You, Carl J. Douglas, Minsheng You

**Affiliations:** ^1^Institute of Applied Ecology, Fujian Agriculture and Forestry University, Fuzhou 350002, China, ^2^Faculty of Life Sciences, Fujian Agriculture and Forestry University, Fuzhou 350002, China, ^3^Key Laboratory of Integrated Pest Management for Fujian-Taiwan Crops, Ministry of Agriculture, Fuzhou 350002, China, ^4^School of Molecular and Biomedical Science, The University of Adelaide, Adelaide SA 5005, Australia and ^5^Department of Botany, University of British Columbia, 3529-6270 University Boulevard, Vancouver, BC V6T 1Z4, Canada

## Abstract

The diamondback moth Genome Database (DBM-DB) is a central online repository for storing and integrating genomic data of diamondback moth (DBM), *Plutella xylostella* (L.). It provides comprehensive search tools and downloadable datasets for scientists to study comparative genomics, biological interpretation and gene annotation of this insect pest. DBM-DB contains assembled transcriptome datasets from multiple DBM strains and developmental stages, and the annotated genome of *P. xylostella* (version 2). We have also integrated publically available ESTs from NCBI and a putative gene set from a second DBM genome (KONAGbase) to enable users to compare different gene models. DBM-DB was developed with the capacity to incorporate future data resources, and will serve as a long-term and open-access database that can be conveniently used for research on the biology, distribution and evolution of DBM. This resource aims to help reduce the impact DBM has on agriculture using genomic and molecular tools.

**Database URL:** http://iae.fafu.edu.cn/DBM/

## Introduction

The diamondback moth (DBM), *Plutella xylostella* (L.), has a worldwide distribution and is one of the most destructive insect pests of cruciferous food crops (1, 2). Annual pest management costs for controlling DBM are approximately US$2 billion; however, if yield losses attributed to insect damage are included, overall estimates escalate to US$4–5 billion (3, 4). Effective integrated pest management strategies rely on the rotation of insecticide sprays, although biological control can be remarkably effective against DBM (2, 3, 5). Overreliance or overuse of insecticides can have negative consequences on DBM control, including rapid development of insecticide resistance (6, 7) and the suppression of beneficial parasitoid populations.

Although a global pest, DBM is also an excellent system for studies on comparative genomics, ecological entomology, morphogenesis, insecticide resistance, migration, phylogenetic evolution and interactions with host plants and/or natural enemies (4). Through sequencing the DBM genome and stage-specific transcriptomes, it is hoped new mechanisms for control will be identified, along with a greater understanding of this insect’s biology. Next-generation sequencing technology has driven major advances in DBM genomics. Baxter *et al.* constructed a sequence-based genetic linkage map of the DBM genome using restriction-site associated DNA sequencing (RAD-Seq) (8). Subsequently, several DBM transcriptomes were sequenced by different organizations (9–11), and, in 2013, the DBM draft genome (Fuzhou-S strain) was publicly released (12). The genome was sequenced using the Illumina platform with a strategy that combined whole genome shotgun data (WGS) with 100 800 sequenced fosmid clones. Recently, the genome sequence of a second DBM strain (Bt-toxin susceptible strain PXS) was generated using the Roche 454 platform and data released at KONAGAbase (13).

Here, we present the DBM genome database (DBM-DB), an organism-specific database that coordinates the genomic resources available for this insect. The database provides researchers with user-friendly access to the genome sequence of the Fuzhou-S strain and related genomic and transcriptomic sequence data. DBM-DB provides a centralized database for the DBM research community, which can access it using a simple and intuitive interface. It also provides a platform for DBM research scientists to manually check gene model annotations and submit information detailing missing genes and/or misannotated genes to our centre (dbm@iae.fjau.edu.cn).

## Database sources

DBM-DB release 1.2 contains transcriptome datasets (Table 1), linkage group information and reference genome scaffolds with alignments of the following: (i) functionally annotated unigenes, (ii) ESTs (NCBI, August 2013) and (iii) a putative gene set generated by Jouraku *et al.* (2013) (KONAGAbase, version 2). Current datasets can be viewed and downloaded at http://iae.fafu.edu.cn/DBM/, and future DBM-DB versions will be generated and released as additional data resources become available.

**Table 1.** Summary of the DBM genome and transcriptome datasets in DBM-DB (version 1.2)

Data set	Number	Percentage^a^ (%)
Genome dataset		
Assembly scaffold (version 2)	1819	
Official Gene Set (version 1)	18 071	
Gene annotation		
SwissProt	12 631	69.90
TrEMBL	14 844	82.14
InterPro	12 877	71.26
KEGG	10 390	57.50
GO	10 745	59.46
Total annotated genes	15 195	84.08
Transcriptome dataset		
Total unigenes	171 262	
Functionally annotated unigenes	38 255	22.34
OGSv1 with RPKM value >1	16 150	89.37

^a^Percentage of the official gene set

### Genome assembly version 2

The Fuzhou-S genome was sequenced using the Illumina platform, and *de novo* assembled with custom software (Rabbit) that incorporated 100 800 fosmid clones and whole genome shotgun data that were both sequenced to a depth of >200X (12). As two divergent haplotypes may be retained within an assembly, we used the Poisson distribution-based *K*-mer statistic (12) to identify allelic regions containing >40% unique *K*-mers. Masking these redundant genomic regions with “n” characters produced the DBM genome assembly version 2. This version release included 1819 scaffolds with an N50 of 737 kb, of which 171 scaffolds were assigned to 31 linkage groups (8, 12). The statistics of our DBM genome version 2 were summarized and compared with the DBM genome as described in KONAGAbase (Table 2).

**Table 2.** Statistics of the DBM genome assembly (version 2) in DBM-DB and KONAGAbase

Data set	Total number	GC %	Size (Mb)	N50 (bp)	Max (bp)
DBM-DB: DBM susceptible (Fuzhou-S) strain (12)					
Scaffolds	1819	38.34%	394	737 182	3 493 687
Contigs (>1 kbp)	31 979	38.34%	334	18 785	202 508
KONAGAbase: Bt-toxin susuceptible strain (13)					
Contigs (>1 kbp)	88 530	38.3%	186	2273	24 960
Degenerate contigs	246 44	38.8%	148	643	12 183
Singleton	106 455	42.0%	31	399	727

### Official gene set version 1

**Figure 1.**
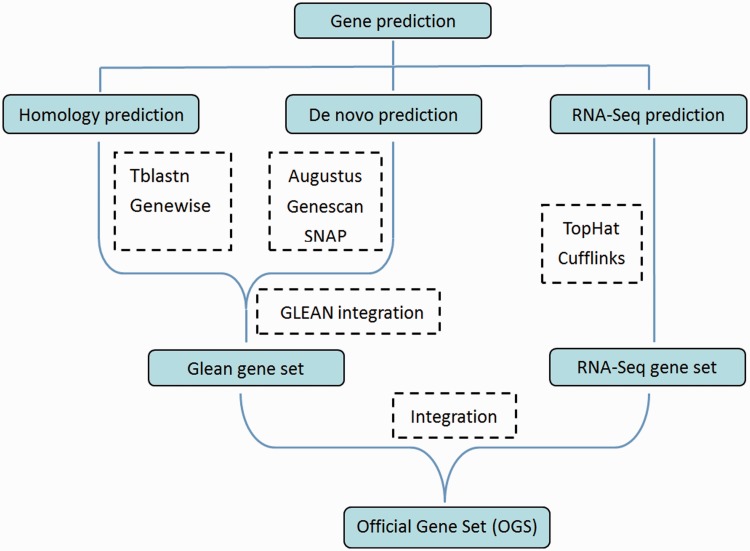
Flowchart of the DBM gene prediction. Software or approaches used for the prediction are shown in dashed boxes.

The DBM whole-genome gene prediction was performed using a combination of approaches (Figure 1). First, genes were obtained using *de novo* prediction with Augustus, Genescan and SNAP tools that generated 19 073 gene objects. Second, homology prediction was conducted against four insect species, including *Drosophila melanogaster*, *Tribolium castaneum*, *Anopheles gambiae* and *Bombyx mori*. Gene models generated through *de novo* and homology prediction were integrated using GLEAN (14); then the transcriptomes generated from RNA-seq were integrated to produce the Official Gene Set version 1 (OGSv1) containing 18 071 genes (denoted as “Px+number”, for example Px018071) (12). The 18 071 predicted DBM genes were annotated using BLAST tools to predict gene function via homology from Swissprot and TrEMBL datasets in the UniProt database. Other gene annotations were conducted using Gene Ontology (GO) (http://www.geneontology.org/) (15, 16), Kyoto Encyclopedia of Genes and Genomes (KEGG) (http://www.genome.jp/kegg/) (17, 18) and InterPro (http://www.ebi.ac.uk/interpro/) (19) databases. As a result, functional information for 15 195 (84.08%) of the DBM OGSv1 was obtained.

### Transcriptome *de novo* assembly

RNA-transcriptome datasets were generated from six different DBM samples, including eggs, larvae, pupae and adults of the insecticide-susceptible Fuzhou-S reference strain and larvae of chlorpyrifos- and fipronil-resistant strains (CRS, FRS). The samples were sequenced and *de novo* assembled into 171 262 non-redundant sequences (unigenes), of which 38 255 were functionally annotated. In OGSv1, 16 150 genes were expressed with the values of RPKM (reads per kb per million reads) ≥1 (9, 12). A summary of the unigenes generated from our transcriptome datasets is presented alongside the unigene dataset described in KONAGAbase (Table 3).

**Table 3.** Statistics of unigenes/ESTs in DBM-DB and KONAGAbase

Data set		Total number	GC %	Size (Mb)	Mean (bp)	Max (bp)
DBM-DB^a^	Egg	70 234	45.3%	31.4	447	14 617
	Larva	69 008	47.9%	24.8	436	20 840
	Pupa	73 194	45.3%	32.5	444	20 840
	Adult	55 943	45.2%	30.1	443	14 617
	CRS	54 869	51.8%	24.9	454	20 804
	RFS	58 565	48.8%	27.4	468	20 840
	Total	171 262	43.0%	93.6	547	23 074
KONAGAbase^b^	NCBI	1722	50.1**%**	1.1	645	16 113
	Midgut	12 406	49.2**%**	6.0	480	879
	Egg	6904	42.6**%**	3.1	446	855
	Testis	16 308	44.2**%**	9.7	446	880
	Larvae (4th)	147 370	42.8**%**	66.5	451	11 311
	Total	84 570	43.0**%**	47.8	564	16 249

^a^Samples at four basic developmental stages of transcriptomes in DBM-DB are from DBM susceptible (Fuzhou-S) strain. CRF and RFS represent the chlorpyrifos- and fipronil-resistant strains, respectively. The total unigenes is the result of clustering and redundancy filtering of six transcriptomes.

^b^KONAGAbase unigenes were assembled the EST/mRNA sequences from NCBI, the ESTs from midgut, egg and testes, and the RNA-seq contigs of the fourth instar DBM larvae.

### Mapping-transcribed sequences to the reference genome

DBM transcriptome data from three diverse sources were separately aligned to the Fuzhou-S reference genome to determine the impact of genetic diversity on the mapping rate. Publicly available ESTs generated from multiple DBM strains (36 907 sequences, NCBI), the putative gene set from Japanese strain PXS (32 800 sequences, KONAGAbase) and functionally annotated unigenes of the Chinese strain Fuzhou-S (38 255 sequences, DBM-DB) were aligned to 1819 genomic scaffolds using GMAP (20) with the designated parameters ≥ 90% coverage and identity. The number of sequences from ESTs, PXS genes and unigenes and those mapped to genomic scaffolds were 29 187 (79.08%), 26 074 (79.49%) and 31 825 (83.19%), respectively. The remaining unmapped sequences were further aligned to fosmid contigs (>5Kb), which resulted in the total mapping rates of 89.19%, 91.75% and 92.29%, for three datasets. When we aligned the ESTs, PXS genes and unigenes to the PXS genome assembly (total of contig, degenerate contig and singleton) using the same method, the mapping rates were 78.49%, 75.86% and 73.36%, respectively (Table 4).

**Table 4.** Statistics of mapping unigenes from Fuzhou-S, PXS and ESTs (NCBI) to DBM reference genomes Fuzhou-S and PXS

Data set	Fuzhou-S unigenes	PXS genes	ESTs
Total number of sequences in dataset	38 255	32 800	36 907
Fuzhou-S genome	31 825 (83.19%)	26 074 (79.49%)	29 187 (79.08%)
Fuzhou-S fosmids (>5 Kb)^a^	3482 (54.15%)	4022 (59.80%)	3732 (48.34%)
Total Fuzhou-S	35 307 (92.29%)	30 096 (91.75%)	32 919 (89.19%)
PXS genome	28 064 (73.36%)	24 882 (75.86%)	28 970 (78.49%)

^a^Fosmid sequences were generated by sequencing 100 800 single colonies to achieve 10× coverage of the genome.

## Database organization

**Figure 2.**
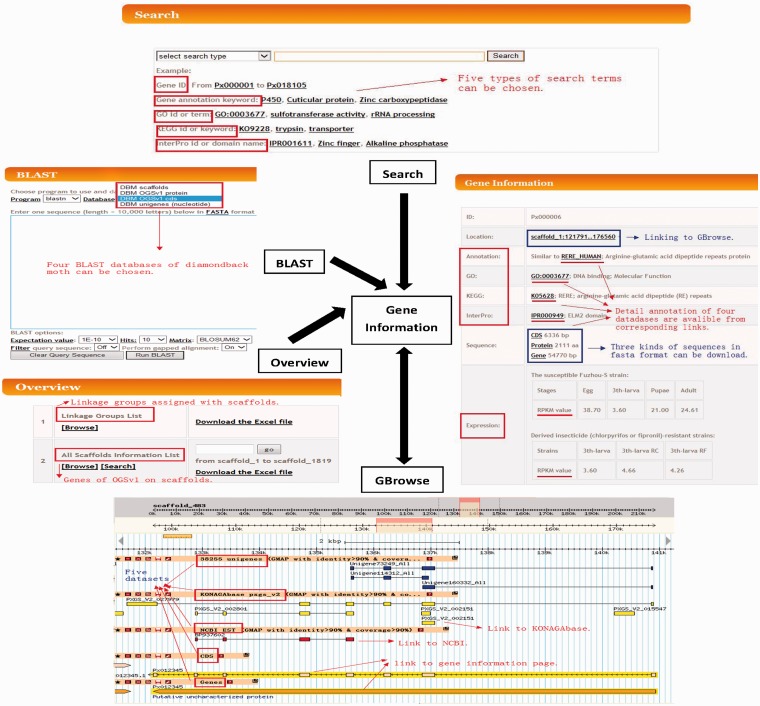
Schematic structure of the DBM-DB. Components including gene information, Overview, Search, BLAST and Gbrowse, and their orientations are presented. Arrows represent the relationships between different components.

The DBM-DB is an extensive online database that catalogues DBM genomic data, published by You *et al.* (12) and He *et al.* (9). It was rationally structured in a user-friendly and web-based mode, containing four primary components of Search, Overview, BLAST and GBrowse, which are interlinked with the Gene Information (Figure 2).

### Gene information

The Gene Information held within DBM-DB and can be readily accessed using the four online components: Overview, Search, BLAST and GBrowse, as shown in Figure 2. A custom PHP script was developed to generate a dynamic HTML page for the overall information of each gene in OGSv1, and the MySQL database language was used as a tool to manage and store the datasets of DBM-DB.

Information on each of the 18 071 OGSv1 genes can be found in the Gene Information component, which contains the scaffold location, Uniprot similarity description, Gene Ontology (GO) term, KEGG pathway annotation, protein domain annotation, CDS sequence, protein sequence and gene sequence (including introns) in FASTA format. In gene expression data generated by RNA-seq are provided as a foundation for the study of gene differential expression. The gene location is linked to GBrowse, which enables gene structure visualization and provides Uniprot, GO, KEGG and InterPro databases accession numbers where available. Each gene structure can be downloaded in GFF3 format from the gene information page. Nucleotide and protein sequences in FASTA format can also be obtained through links provided (Figure 2).

### Overview

A total of 171 scaffolds were assigned to 28 of 31 linkage groups, which represent different chromosomes (8, 12). The Overview component in DBM-DB contains information listing the scaffolds that have been assigned to specific linkage groups. The Linkage Groups List option enables users to browse all scaffolds with linkage group assignment, and the All Scaffolds Information List enables users to browse or search for specific scaffolds (Figure 2). Furthermore, the All Scaffolds Information List provides data outlining the number of contigs in a specific scaffold, the gap rate, GC content and a list of genes on each scaffold. Each of the gene IDs and locations are linked to the Gene Information and GBrowse components, respectively.

### Search

The search component allows users to retrieve gene information of interested by inputting specific codes and/or keywords accordingly. Five types of search terms are available: Gene ID ranging from Px000001 to Px018105 (a total of 18 071 genes were included in the final OGSv1), annotation keyword, GO ID or term, KEGG ID or keyword and InterPro ID or domain name. Under the selection box, several examples for each of the five search types have been provided to help users navigate this function. Search results provide the number of related genes and their gene IDs. By clicking the gene ID hyperlinks, users can be navigated to the core component or the Gene Information.

### BLAST server

In order to facilitate sequence homology searches, we implemented the basic local alignment search tool (BLAST) (21). Users can search against DBM sequences including genomic scaffolds, transcriptomic unigenes and OGSv1 CDS or proteins. The scaffolds, unigenes and gene CDS sequences can be searched using nucleotide sequences with blastn or tblastx options. Blastp and blastx can also be conducted to search against the database of protein sequences using protein and nucleotide sequences, respectively. In addition, we developed a set of PHP scripts to call the program of BLAST and customize BLAST output, on which the subject ID of DBM-DB is linked to the corresponding Gene Information component.

### Genome visualization

The genome browser (GBrowse) is a tool that integrates databases and interactive web pages for visualizing genome information (22). GBrowse can display a specific DBM scaffold with the following: (i) the corresponding annotation and structure of our OGSv1 genes; (ii) homologous, functionally annotated unigenes; (iii) DBM ESTs from NCBI and (iv) the putative PXS gene set from KONAGAbase. Users can therefore view and navigate genomic scaffolds, which include information for gene annotations, gene structure (based upon OGSv1), ESTs and PXS genes. This enables users to simultaneously view independent datasets when assessing gene models. CDS and gene tracks are linked to the Gene Information component, and external links to GeneBank and KONAGAbase are available by clicking the EST or PXS gene alignment tracks (Figure 2).

### Download page

In the download HTML page, both FTP and HTTP links are provided for users to download entire datasets, as required. The FTP site of DMB-DB (ftp://iae.fafu.edu.cn/pub) contains genomic scaffolds (draft genome version 1 and version 2) and predicted OGSv1 gene sequences in FASTA format and gene structure in gff3 format. Gene annotation is also provided, including gene functional description, KEGG, GO and InterPro domain. DBM transcriptomes from egg, larvae, pupae and adult tissues are available for download, along with the combined *de novo* assembled transcriptome (All-Unigene assembly version 1) in FASTA format plus their expression information. In addition, some useful files are available, which include alignments between scaffolds/fosmid contigs and different DBM sequences (ESTs, PXS genes, functionally annotated unigenes).

## System implementation

**Figure 3.**
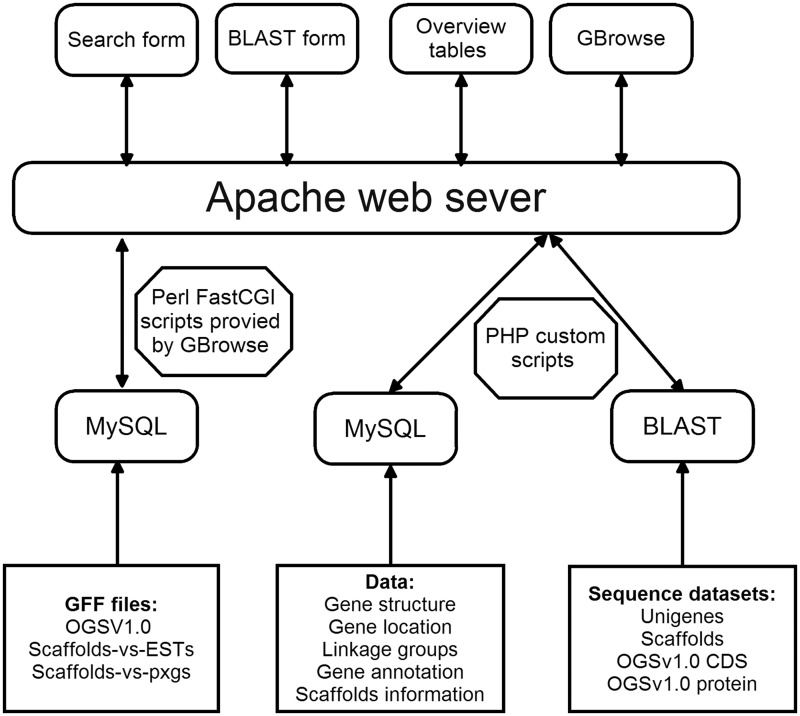
Architecture of the DBM-DB.

DBM-DB was developed under the Linux system using several common software packages including PHP, Apache web server, MySQL database management and Perl FastCGI (Figure 3). Several custom PHP scripts were developed to make the database flexible, interactive and intuitive so that users could readily access and obtain the information they need either for molecular analysis or practical application. In addition, the generic Genome Browser (GBrowse) package, a component of the Generic Model Organism Project (GMOD), was used for genome data visualization, which allows users to obtain the information on gene structures based on the DBM genome assembly. In order to search against the DBM genome, the local BLAST tool was installed in the DBM-DB system.

## Future work

DBM-DB provides a large-scale set of the genomic data and a convenient tool for further research on genomics, genetics and molecular biology of *P.xylostella* and other species of insects. This database was designed with the room to accommodate and house future data that will be generated, and efforts will be made to regularly update and upgrade the data resources. We are aiming to improve access of both transcriptome and genome data in the future. Future resources to be developed include digital gene expression profiling of different developmental stages or tissues, data supporting microRNAs expression and the meta-genomics of DBM midguts. Genome resources will be updated when appropriate, including improving scaffolds, assigning additional scaffolds to chromosomes using genetic mapping, more precise gene prediction and functional annotation and the upcoming information on DBM phylogeography. Further, DBM sequences from NCBI database as well as DBM-related publications will also be integrated into DBM-DB. To further support the capability of DBM-DB to serve the research community, new web tools are being developed to allow more efficient and effective use of the DBM genomic information-housed DBM-DB.
